# Book Review

**Published:** 1895-06

**Authors:** 


*Syllabus of Lectures on Human Embryology.* By WALTER PORTER MANTON, M.D. Pp. vi., 125. Philadelphia: THE F. A. DAVIS CO. London: F. J. REBMAN. 1894.—This book is not intended to be an independent text-book of embryology, but to be supplemented by notes taken in the class-room. For this purpose the work is interleaved with blank sheets. The work is too small to allow the subject to be treated in other than an imperfect manner; but it gives an outline of embryology, some practical hints on the preparation of specimens, and a full glossary of terms.

*Text-Book of Hygiene.* By GEORGE H. ROHÉ, M.D. Third Edition. Pp. x., 553. Philadelphia: THE F. A. DAVIS CO. London: F. J. REBMAN. 1894.—This edition has been carefully revised and, in most instances, brought up to date. While it can hardly supplant, for English readers, the many excellent text-books already in use, the style is interesting, and the information practical. It is useful, also, to be able to compare, for example, the American quarantine regulations with those in force in this country; and the chapter on this subject (contributed by Drs. Wyman and Geddings) is carefully written. Analytical questions appended to each chapter render the work more convenient as a text-book for students.

*Helps in Sickness and to Health.* By HENRY C. BURDETT. Pp. xv., 484. London: THE SCIENTIFIC PRESS, LIMITED. 1894. —This book is really a second edition of the author's works entitled *Helps in Sickness* and *Helps to Health.* It affords a large amount of well-selected and sound information of a kind frequently desired by the householder. In addition to its chief contents, there is a concise summary of the sanitary powers and duties of the citizen; and in Part III. are given full particulars of all institutions in England and Wales founded for the relief of sickness or bodily infirmity. The variety of the information afforded should make this volume useful as a reference, not only to the householder, but also to the medical attendant.

*The Dynamics of Life.* By W. R. GOWERS, M.D., F.R.S. Pp. 70. London: J. & A. CHURCHILL. 1894.—The title of this address, which was delivered before the Medical Society of Manchester on October 3rd, 1894, is a trifle misleading: it is not Life, but the processes associated with it and which it controls that are considered. Dr. Gowers is careful to make this clear, and wisely disclaims any attempt to unravel the mystery of our vitality. He warns his hearers that he must ask for their close attention; and the advice is needed, for the reasoning must be carefully followed to be grasped. Briefly, his argument is this: The various kinds of activity which take place in our body are due to chemical energy—at first *latent*, then *actual*. This energy is a form of minute motion—molecular, atomic, and sub-molecular (that is, between molecules or atoms or the "radicals" of which molecules are composed). This motion tends to dissipate atoms and molecules, but is held in check by the force of chemical attraction. The normal and abnormal action of muscle and nerve are taken as examples to illustrate this point. When a stimulus, which is a form of molecular motion, travels down a nerve to a muscle, the added movement releases the muscle molecules, which are free to move in the direction of greatest attraction—that is, towards the oxygen in the surrounding plasma. The "rush" to the surface of the fibrillæ causes the transverse thickening, and "molecular" is changed into "massive" movement. The impulse in the nerve-fibre comes, not from the nerve-cells, but from the mesh-work of axis-cylinder fibrils near them. In the complex, unstable compounds of the nervous system, the inherent molecular motion is nearly equal to the restraint, and the slightest stimulus may therefore be sufficient to produce violent change, as in a finely adjusted balance the equilibrium of the heavy weights may be upset by a grain. In epilepsy and kindred "explosive" diseases the molecular motion during the convulsion *exceeds* the restraint. These thoughts are expressed as clearly as the nature of the subject permits, and useful similes are employed here and there, but the reader feels that the ground he walks on is uncertain and near the confines which limit our knowledge.

*Transactions of the Clinical Society of London.* Vol. XXVII. London: LONGMANS, GREEN, AND CO. 1894.—This volume is full of papers of great interest and value. The Clinical Society is a very flourishing one, and has lately added much to the interest of its meetings by setting apart certain evenings for exhibition of patients. In his presidential address at the opening of last winter's session, Mr. Hulke observed that the progress may be discerned not only in the large increase in the number of members, but also in the quality and thoroughness of the communications made to it in recent years. This is to be seen in most of the papers published in the present volume. It would be impossible in a brief review, such as this must be, to allude to the various papers, but we may mention that many of the topics at present engaging the attention of the profession are illustrated by reports of important cases. The surgical treatment of perforated typhoid ulcers and suppurative general peritonitis may be referred to as examples.

*Saint Thomas's Hospital Reports.* Vol. XXII. London: J. & A. CHURCHILL. 1894.—These reports are as usual of high interest and value. They record the deaths of two members of the teaching staff. Dr. W. B. Hadden, cut off at the early age of thirty-seven, at the outset of what promised to be a most distinguished career, was best known for his researches in the field of neurology. His upright, amiable character, and his readiness at all times to help others, endeared him to all with whom he was brought in contact, and his early death is mourned by a large circle of friends. Mr. George Rainey died at the ripe age of eighty-four, after nearly sixty years' work at the Hospital. As a teacher of anatomy he was in his day unsurpassed. He was the author of many original papers of much scientific value, and, besides being an untiring worker and teacher, was a man of remarkable personal character, who made a great impression upon his pupils and contemporaries. The volume contains, amongst many other valuable contributions, a useful practical paper by Mr. H. H. Clutton on the treatment of hallux valgus. Dr. Sharkey describes and figures two remarkable cases of acute bronchiectasis. Dr. Cullingworth's able contribution, which gives a full report and analysis of twenty cases of uterine myoma treated by removal of the uterine appendages, deserves, and will doubtless receive, careful study from all workers in gynæcology. The reports of the Medical and Surgical and other departments of the Hospital are very clearly and carefully made out; they give a complete record of all the Hospital work, and a more detailed account of the most important cases. The Medical School Calendar and Prospectus, which gives a complete list of all Old St. Thomas's students, ends the volume.

*Catalogue of Surgical Instruments and Appliances manufactured by Arnold & Sons.* Pp. xlvii., 848. London. 1895.—This handsome volume contains no less than 3,230 illustrations of surgical instruments and appliances, together with a price-list, and forms an invaluable work of reference for the operating surgeon or the author of a surgical text-book. It is furnished with a complete index, and altogether reflects great credit upon the enterprising firm who have published it.

---

We have received from Dr. W. J. Morton, of New York, a short but interesting account of the claims of his father, Dr. W. P. G. Morton, of Boston, as the "inventor" and "revealer" of anæsthesia. The occasion for the publication of this claim is the inclusion of Dr. Morton among the fifty-three "Immortals" of Massachusetts whose names are inscribed round the dome of the new chamber of the House of Representatives. No one who has studied the history of anæsthesia will dispute the right of conferring this high honour on Dr. Morton's memory, but some looseness has crept into the expressions of praise engraved elsewhere which we think only right to correct. Dr. Morton was not the "inventor of anæsthesia" or "the inventor and revealer of anæsthetic inhalation," as the inscriptions on his monuments wish us to believe. To him belongs the credit of introducing ether as an anæsthetic agent to the medical profession, but it is a mistake to credit him with being the "inventor of anæsthesia" or of "anæsthetic inhalation," for anæsthesia had been produced many years previously by the inhalation of other agents. Nearly fifty years before, Sir Humphry Davy had shown that nitrous oxide would produce insensibility, and Horace Wells, of Hartford, Conn., had employed it at least two years before Dr. Morton used ether. Even the property of ether to produce anæsthesia had been proved by Dr. W. C. Long, of Jefferson, Jackson County, Galveston, who painlessly excised a tumour from James Venables in March, 1842; Dr. Morton's first operation—one for extraction of a tooth—was done on September 30, 1846. By erecting two monuments to his honour —one over his grave in Mount Auburn Cemetery, the other in the Public Gardens—Boston has honoured, and justly too, the memory of the illustrious citizen whose first public demonstration of the anæsthetic properties of ether was made on October 17, 1846, at the Massachusetts General Hospital. On these monuments are inscriptions containing the words quoted above which, for the reasons we have mentioned, appear to us to be infelicitous in expression, if not inaccurate in fact. The word anæsthesia was the invention of Dr. Oliver Wendell Holmes. Like many of the greatest benefactors to mankind, Dr. Morton's honours are posthumous, for we read: "He died poor, and '*he became poor in a cause which has made the world his debtor.*'"

MM. J.-B. Baillière et Fils, of 19 Rue Hautefeuille, Paris, have just issued a new general catalogue of books relating to medicine and the allied sciences. It is a useful work of 112 closely-printed pages, which the publishers will be glad to send to anyone applying for a copy.

